# Using a World Health Assembly simulation to explore undergraduate students’ perceptions and confidence in analyzing complex global health challenges: A mixed-methods evaluation

**DOI:** 10.1371/journal.pgph.0002792

**Published:** 2025-11-03

**Authors:** Ahmad Firas Khalid, Megan A. George, Clarissa Eggen, Aaranee Sritharan, Faiza Wali, A. M. Viens

**Affiliations:** 1 School of Global Health, York University, Toronto, Ontario, Canada; 2 Dahdaleh Institute for Global Health Research, York University, Toronto, Ontario, Canada; 3 Faculty of Medicine, University of Toronto, Toronto, Ontario, Canada; 4 Global Health Strategy Lab, York University, Toronto, Ontario, Canada; New York University Grossman School of Medicine, UNITED STATES OF AMERICA

## Abstract

Traditional didactic teaching approaches fall short of adequately supporting diverse student learning styles. Complementing didactic teaching approaches with simulation-based experiential learning can bridge the gap between theoretical knowledge and practical application. However, few studies have rigorously examined the outcomes of this approach in global health education and training. This study describes participants’ self-reported experiences with the World Health Assembly Simulation (WHA SIM), a complex hybrid simulation consisting of a three-day educational exercise, including a tabletop exercise followed by live-action role play, designed to simulate practical global health governance settings. We conducted a descriptive, sequential exploratory study between September 2022 and July 2023, beginning with an anonymous pre-simulation survey among undergraduate students in the Faculty of Health at York University, followed by qualitative post-simulation interviews. The survey included questions on participants’ background, comfort, and confidence in key practice skills and experiences with simulation-based learning. Data was analyzed using simple descriptive statistics for the quantitative data and a framework analysis for qualitative data. Among 39 survey respondents, 18 were interviewed. Participants for interviews were selected through snowball sampling to ensure diverse perspectives. Participants reported feeling more confident in a range of skills, including research capabilities, critical analysis, time management, and organizational effectiveness. They also described increased confidence in interpersonal communication, public speaking, networking, collaboration, and leadership. Several participants reflected on gaining a deeper appreciation of complex global health issues and noted the simulation provided valuable career-relevant insights. Findings highlight the value of simulation-based experiential learning as perceived by undergraduate students in health and science-related degree programs. While this study does not objectively measure knowledge acquisition or skill mastery, it illustrates how the WHA SIM can support confidence and reflective learning in global health education and training contexts.

## Introduction

The escalating frequency of health and humanitarian crises has significantly fueled students’ engagement with global health topics and institutions, such as the World Health Organization (WHO), which is responsible for addressing these intricate challenges [1–4]. Amidst this heightened interest, exacerbated by events like the COVID-19 pandemic, simulation-based experiential learning stands out as a transformative educational approach. It offers students a tangible way to apply theory to real-world dilemmas; bridging classroom learning and practice. However, a knowledge gap exists in how to effectively develop and implement these simulations to enhance students’ analysis and problem-solving skills concerning complex global health issues [4–7]. Previous studies vouching for experiential learning’s efficacy often lack rigorous validation, leaving room for enhancement in this educational approach. Moreover, the deployment of simulations, especially in the realm of public and global health education, is a relatively untapped but burgeoning strategy.

In addressing this gap, we apply an educational tool termed the World Health Assembly Simulation (WHA SIM). Our WHA SIM is designed to mimic the WHA’s critical role as the leading decision-making body in global health. The WHA SIM seeks to enhance knowledge and confidence around collaborative governance approaches involving multi-sectoral and multi-jurisdictional global challenges, such as those found in the Sustainable Development Goals (SDGs). It enables the generation and testing of innovative and feasible solutions on the simulation theme in the form of research briefs, debates, and formal resolutions. This endeavor enhances the educational toolkit with an immersive experience while better preparing students for real-world challenges, thereby improving the overall quality of global health education and training activities.

According to classic experiential learning theory, individuals gain knowledge through concrete experience and abstract conceptualization, reflective observation, and active experimentation [8,9]. In other words, the experience of engaging in the learning activity can lead to new knowledge, learning, and skills development. Simulation-based learning in global health education represents an innovative approach to experiential learning, designed to extend beyond traditional classroom boundaries. This method facilitates the exploration and development of practical, effective strategies to address the multifaceted challenges inherent in global health, preparing students with essential skills required in the real-world job market, including writing (e.g., policy briefs), communication (e.g., presenting key points), negotiation (e.g., managing agreements and disagreements), leadership (e.g., meeting deadlines for resolutions), and team building (e.g., working towards consensus) [3].

This study has three primary objectives: 1) To describe the development and implementation of the WHA SIM; 2) To document participating students’ self-reported confidence, experiences, and perceptions following the WHA SIM; and 3) To explore participants’ reflections on how the WHA SIM may influence their perceived readiness for leadership roles in global health.

## Methods

### Ethics statement

Ethical approval was obtained from the Office of Research Ethics (ORE) at York University. Certificate #: e2022-401. We obtained written and verbal consent from all participants.

### Design

**Fig 1:**
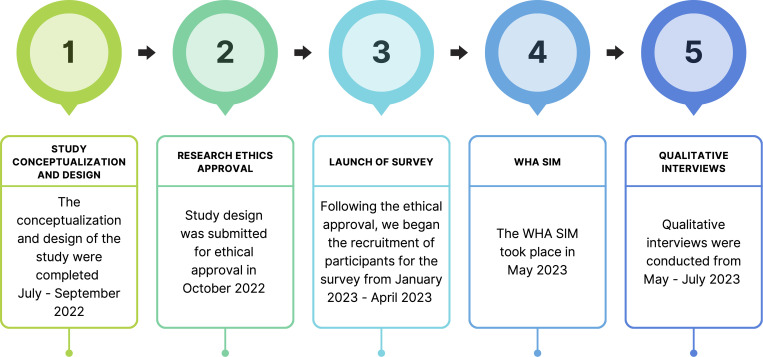
Timeline and activities of research study.

We used a sequential exploratory study design, combining descriptive quantitative and qualitative research approaches to understand participants’ perceptions of simulation-based experiential learning and how it influences their confidence in analyzing complex problems and developing innovative solutions (Fig 1). Initially, we conducted general and targeted needs assessments through an anonymous survey of undergraduate students at York University in Toronto, Canada, across diverse programs of study and years (S1 Table). While this sample does not represent the full diversity of global health professionals, it reflects a multidisciplinary group engaged in relevant academic fields and supports interprofessional education within the study’s context. The survey aimed to capture students’ self-reported views and experiences with simulation-based experiential learning. Subsequently, insights from the survey informed qualitative interviews with students who participated in the WHA SIM, providing an in-depth understanding of participants’ reflections on their confidence, perceived skill development, and suggestions to enhance the delivery of simulation-based learning. These findings were further supported by observations from the principal investigator (Khalid, AF) during the WHA SIM.

In designing the pedagogical teaching strategy, we adopted an educational approach emphasizing experiential learning through preparatory materials, interactive sessions, and reflective practices to support the simulation’s objectives. The WHA SIM consisted of a rigorous three-day exercise, including a tabletop exercise—discussion-based simulations to evaluate responses to hypothetical global health scenarios—followed by live-action role play for hands-on experience.

The WHA SIM was structured to provide an immersive learning experience, mirroring real-world global health negotiations. Activities included drafting position papers, committee sessions, and resolution ratification, reflecting the actual workings of the WHA. Integration of technical experts and real-world scenarios enriched the simulation, ensuring an engaging educational experience representing global health governance complexities.

**Fig 2:**
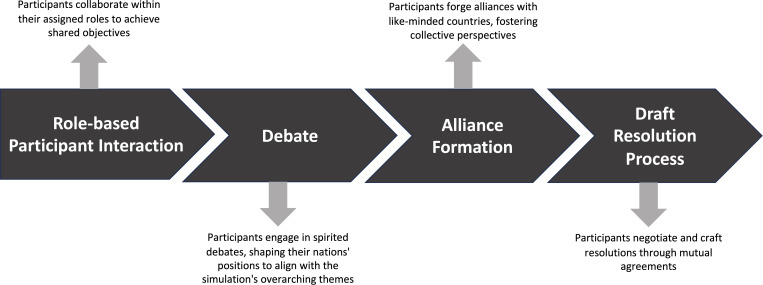
The four components that comprise a WHA Simulation.

The 2023 WHA SIM theme, “Building Global Solidarity for Worldwide Health Security,” focused on urgent health emergencies like pandemics, aligning with previous simulations such as the 2021 Model WHO Simulation Week in London, organized by the UK Network for Model WHO Simulations, as well as Sheffield World Health Organization World Health Assembly simulation, which has run WHA SIMs from 2018. To ensure inclusivity, participation was free, funded by grants to cover design and implementation costs. Day one, delivered virtually, equipped delegates with essential skills, including understanding WHA SIM protocols, crafting position papers, and mastering debate strategies. Day two transitioned to in-person activities at York University, featuring an inaugural ceremony, a panel discussion with global health luminaries, committee sessions on key issues, and side events to enhance professional skill development. The final day emphasized collaborative decision-making through creating and ratifying resolutions. The event concluded with plenary discussions, closing remarks, and an awards ceremony recognizing students’ contributions. Dr. Tedros Adhanom Ghebreyesus, WHO Director General, shared remarks recognizing the WHA SIM’s educational value in advancing global health learning (Fig 2).

### Research participant recruitment and sample size

A two-stage sampling approach was used to identify and recruit key informants [10,11]. The first stage included recruiting students from various disciplines in the Faculty of Health and the Faculty of Science. To recruit these participants, we created a York University centrally supported learning management system (eClass) announcement distributed to faculty members to share on their course eClass sites. We also sent out emails to faculty program administrators to share the advertisement of the study with students via email. Finally, the research team created a video to invite students to participate in the study, which was shared on the Faculty of Health and the School of Global Health Twitter/X page. The link to the survey was sent to the interested students who would contact us after checking their eligibility and getting their consent. To incentivize students’ participation in our survey, we offered them a raffle for an Amazon gift card. The second stage involved respondent-driven sampling by which students in the first stage were asked to identify any additional informants. We planned to sub-sample participants for the semi-structured interviews from the pool of students who had responded to the survey and were enrolled in the WHA SIM. Given that our goal was to gain information from students who were readily available and ‘convenient’ students to access, we anticipated a sample size of 35 – 50 research participants. We recruited 39 participants from our first stage of sampling, and 18 additional participants were identified through snowball sampling for the qualitative component of the study. For this study, we conducted interviews with 18 participants, reaching a saturation point after 15 interviews, where no new themes or insights were emerging. However, we continued with the remaining interviews to ensure the robustness and credibility of our findings.

### Data collection methods

#### Phase one: Quantitative study.

The pre-simulation online survey was administered immediately after students registered for the WHA SIM. The survey was in English and was administered using Microsoft Office 360 Forms. The survey questions were developed with the intent to gather insight and gauge student interest and preparedness to participate in the WHA SIM. The survey questions consisted of three parts: (1) questions focusing on participants’ background and demographic characteristics, (2) questions that captured participants’ comfort and confidence in key practice skills, and (3) questions around participants’ experiences and preferences with simulation-based learning.

#### Phase two: Qualitative study.

Semi-structured interviews were conducted via Zoom within weeks following the WHA SIM to encourage participation. Interviews, lasting approximately 30 minutes, explored participants’ subjective experiences with the simulation, focusing on perceived skills gained, confidence in addressing global health challenges, and suggestions for improving the simulation’s educational value. Interviews also invited reflections on readiness for leadership roles, acknowledging that these are perceptions rather than measured outcomes. Recordings were transcribed verbatim by research team members (MG, CE, AS, FW), with removal of identifying information. Transcripts were used for thematic analysis to identify participant perspectives and insights.

### Data analysis

A descriptive and exploratory approach to data analysis was employed. For the survey, quantitative data was summarized using simple descriptive statistics (numbers, percentages, frequencies, and cross-tabulation). Analysis focused on describing participant characteristics, their self-reported experiences with experiential learning, and their attitudes towards participation in simulation-based learning. No inferential or causal analyses were conducted. Descriptive statistics were calculated for participant background, prior experiential learning, and attitudes toward simulation-based learning. For the semi-structured interview data, we used a deductive framework analysis approach to explore participants’ reflections and perceptions [12,13]. Framework analysis, a qualitative method suitable for studies with specific questions and limited time frames [12], enabled us to interpret participants’ perspectives on simulation-based learning [13]. The process followed five key steps: familiarization (i.e., immersing ourselves in collected data making notes of key ideas and recurrent themes), identifying a thematic framework (i.e., recognizing emerging themes), indexing (i.e., using NVivo to identify sections of data that correspond to particular themes), charting (i.e., arranging identified sections of data into table exhibits), and mapping and interpretation (i.e., analyzing key characteristics from the exhibits) [13]. Each interview transcript was assigned a unique identifier to ensure anonymity while linking quotations to themes for clarity and reliability.

## Results

We first outline the detailed planning and execution process of the WHA SIM (Fig 1), followed by an overview of our participants. Subsequently, we present an analysis of participants’ self-reported experiences with global health simulation-based experiential learning, focusing on their perceptions of skill development and preparation for future leadership roles in global health. We highlight the key themes that emerged, illustrating the skills participants perceived they developed through engagement in the WHA SIM.

### Participants’ characteristics

#### Phase 1: Quantitative results.

**Views and experiences in simulation-based experiential learning:** Participants’ experiences and perceptions of simulation-based experiential learning were explored in the study (S2 Table). Regarding prior involvement in simulation-based learning, only 33.33% of participants had engaged in such activities, while 51.28% had no previous involvement and 15.38% were uncertain. Awareness levels of simulation-based experiential learning were higher, with 66.67% of participants knowledgeable about this educational approach, 17.95% unaware, and 15.38% only somewhat aware. Familiarity with the WHO’s WHA was prevalent, with 76.92% having heard of it, though only 17.95% were knowledgeable about its purpose, and 5.13% had never encountered it. The belief in the necessity of simulation-based learning for their education was strong among participants, with 59.97% in agreement, though a notable 38.46% remained unsure, and a minimal 2.56% disagreed. Inquiry into the integration of any form of simulation-based learning during their degree revealed mixed responses: 38.46% confirmed its presence, 41.03% denied it, and 20.51% were uncertain. Assessing the perceived efficacy of simulation-based learning in preparing participants for real-world experiences yielded positive views: 48.72% felt it very effective and 51.28% deemed it somewhat effective, with none of the respondents labeling it as ineffective in any degree.

**Comfort and confidence in key practical skills:** Participants’ self-assessment survey revealed high self-reported confidence in communication (92.31%) and problem-solving skills (86.84%), but less comfort interacting with leadership (66.67%) and task prioritization challenges (61.54%). While 97.44% recognized opportunities to improve time management and critical problem-solving, only 66.66% felt ready for professional practice post-graduation, highlighting a perceived gap in career preparedness.

**Preparing for real-world career skills:** In assessing participants’ perceptions of the impact of simulation-based learning on real-world career skill preparation (Table 1), the majority acknowledged substantial benefits across multiple competencies. Participants found simulation-based learning “very helpful” in familiarizing themselves with work environments (69.23%), tackling global health challenges (74.36%), and enhancing critical skills, such as delegation, knowledge application, and critical thinking (74.36%). Similarly, understanding role expectations and legal matters (69.23%), effective communication (64.10%), and cultural respect (56.41%) were areas where simulations were considered significantly advantageous. Though the perceived helpfulness slightly diminished in aspects like managing workloads (53.85%), technology use (53.85%), and recognizing when to seek help (43.59%), the majority still recognized the value. Collective decision-making (76.32%) and leadership skills (78.95%) were among the most positively perceived areas.

**Table 1:** Overview of participant evaluations on the influence of simulation-based learning in perceived enhancement of real-world career skills across three core competency areas.

Professional Development	Interpersonal Skills	Knowledge and Understanding
• Communication • Confidence • Preparation for future career • Leadership	• Teamwork • Critical thinking • Collaboration • Compromise • Organization	• Enhanced understanding of processes • Exposure to complex problems • Making intellectual inquiries • Highlighting knowledge gaps • Retention through applied learning

#### Phase 2: Qualitative results.

**Participants’ reflections on knowledge and practical understanding:** The analysis of 18 semi-structured interviews revealed participants’ perceptions of deepening practical understanding and knowledge through their involvement in the WHA SIM. These are participant reflections and are not objective measures of knowledge gain but provide useful context surrounding activities that can be used to support knowledge gain. Participants described enhanced comprehension of processes, consolidation of classroom concepts, exposure to complex problems, identification of knowledge gaps, and improved retention through applied learning. One participant remarked: *“Interacting with various fields and applying knowledge in real-world contexts made me realize my extensive learning over four years... I’ve become a specialist with substantial expertise, which is key for career success.”*

Participants also reported increased intellectual engagement. One participant shared, “*Being around people who ask questions and engage, I learned to do the same... asking the right questions can build valuable mentor relationships.”*

Furthermore, participants described the WHA SIM as encouraging open, creative problem-solving. A participant noted: *“The absence of a rubric allowed for creative, collaborative solutions, free from strict guidelines.”* These reflections illustrate how participants perceived the simulation as fostering confidence and practical skills.

**Interpersonal skills and collaboration:** Many participants reported perceived improvements in teamwork and compromise, essential for problem-solving and leadership. One participant shared,

*“Collaborating with people who may lack certain background knowledge taught me how to balance giving feedback without coming across as critical or taking control of the project. It’s about expressing ideas clearly without hurting anyone’s feelings and ensuring the final product is something we’re all proud of.”*

Although the study did not include validated objective assessments of critical thinking performance, several participants reported increased confidence in their critical thinking abilities following the WHA SIM. One participant reflected, “*The simulation made me think out of the box when I’m creating solutions for a certain problem*.”

The simulation also fostered a solution-driven, collective problem-solving environment. A participant observed,

*“Working in smaller groups allowed us to brainstorm ideas collaboratively, resulting in more innovative solutions. Someone would suggest an idea, and others would build upon it, leading to the evolution and improvement of our concepts over time.”*

Participants described gaining insights into the practical challenges of global health work, such as change management and stakeholder engagement. One stated, “*I think that’s one of the things that the WHA SIM helped with is the practicality of how a lot of this work is being done behind the scenes*”, while another reflected on the complexity of convincing diverse stakeholders, “*It’s about like changing entire populations’ perspectives, which requires a lot more patience and intentionality than trying to convince one person at a time.*”

Additionally, the simulation inspired some participants to see themselves as change agents, particularly regarding the SDGs. One participant felt the experience was instrumental in inspiring new ideas and potential research directions, asserting,

*“that simulation will help inspire new ideas... we can help be the agents of change that help facilitate issues, especially the SDG goals. [...] I think the simulation is good to have to find solutions for the world’s issues.”*

**Professional development:** Participants identified the WHA SIM as a valuable learning opportunity contributing to their professional growth. The immersive nature helped participants “*think on their feet,”* contrasting with *traditional* classroom presentations or essays. One participant noted: “[...] *you’re building that skill in a non-judgmental setting*”.

The simulation also offered a realistic preview of potential career paths and allowed participants to assess and reflect on their competencies. One shared:

*“I think just from like picturing myself working later on. As someone who’s always said they would love to work in the World Health Organization, I was like okay it would be good to participate in this kind of opportunity and just get a sense for what it would feel like and the exact skills that are needed just to see if what I currently have would be enough or what I need to improve on.”*

This reflection highlights the simulation’s role in career preparation and self-assessment, further supported by another participant: *“[...] that helped me to identify my weaknesses and actually see it not even like just I know, but I’ll be in this part and seeing that I actually need to work on my articulation*”.

Participants also reported that the simulation increased their confidence and preparation for their future roles in global health, offering practical insights into the complexities of global health leadership and diplomacy. As one participant put it, it was about the “confidence that it gave me.”

Furthermore, the WHA SIM environment nurtured leadership skills in ways traditional classrooms could not. Participants took on active leadership roles, navigating challenges, and decision-making without a predefined path. One participant reflected on this growth: *“The main thing is how to be a leader. [...] But this was new and so you had to step up [...]”.*

## Discussion

Our study examined participants’ perceptions of the extent to which simulation-based experiential learning enhances students’ self-reported abilities to analyze complex issues and devise innovative solutions, particularly within the realm of global health. A significant finding was that despite varied exposure to simulation-based experiential learning, there was broad recognition of its value, with participants reporting its role in bridging theoretical knowledge and practical skills. Participants perceived the WHA SIM as enhancing confidence, creative problem-solving, and collaboration in addressing complex global health challenges. The simulation environment also appeared to bolster participants’ confidence in communication, problem-solving, and leadership, despite some expressing initial discomfort when taking on leadership roles and acknowledging challenges in task prioritization.

In terms of implications for preparing students for future leadership positions in global health, the study highlighted the potential role of simulation-based experiential learning in professional development. The WHA SIM was described by participants as particularly impactful, providing a realistic preview of potential career paths and serving as a platform for participants to evaluate and reflect on their skills, contributing to perceived career preparedness. Participants reported enhancements in competencies including public speaking, critical thinking, and leadership, attributing this growth to the unique challenges presented by the simulation. The immersive experience allowed participants to envision themselves in future careers, especially those aspiring to positions at organizations like the WHO, offering a practical perspective on the skills they may need to develop or refine. Participants reported that the simulation provided practical insight into global health leadership and diplomacy, clarifying the skills needed for future leadership roles.

### Findings in relation to other studies

#### Skill development and professional preparedness.

The skill sets honed by participants during the WHA SIM, encompassing writing, communication, leadership, and teamwork, reflect the competencies sought in the contemporary job market. These reported abilities resonate with Kolb’s experiential learning theory, underscoring the necessity to diminish the skill gap between academic learning and professional requirements [8]. Participants’ survey responses highlighted increased confidence in communication (92.31%) and problem-solving skills (86.84%), supporting Kolb’s theory. Notably, the simulation’s real-world fidelity, whether in mirroring the actual WHA procedures or simulating workplace challenges, was pivotal. This adherence to realism not only enriched the learning experience but also aligns with the National Society for Experiential Education’s best practices [14]. The experiential approach’s veracity appeared to motivate participants, who perceived the knowledge gained as relevant and transferrable to their prospective careers [15].

#### Interactive engagement.

The centrality of active engagement in academic achievement is well-documented, with studies, such as Hake (1998), showing that interactive methods in an introductory physics course nearly doubled the conceptual comprehension scores relative to traditional lectures [16]. While our study did not evaluate test scores, the WHA SIM’s interactive nature reportedly enhanced participants’ engagement and application of concepts. Interview feedback indicated participants found hands-on simulation experience significantly improved their reported understanding and retention of complex global health issues.

#### Comparative insights.

Our findings complement those of similar studies in the realm of simulation-based learning across disciplines. For example, Elias (2014) explored how simulations enhanced students’ understanding of European Union (EU) politics and found improvements in teamwork and public speaking abilities, as measured through both knowledge tests and confidence measures [17]. In contrast, our study relied solely on self-reported measures collected after the simulation; thus, direct comparisons of knowledge gain between the two studies are not possible. Nevertheless, both studies report improvements in participant confidence regarding public speaking and teamwork. These parallels suggest that simulation-based learning may be a versatile pedagogical approach across fields, including healthcare, public health, and policy, particularly for fostering skills that participants perceive as valuable for decision-making and collaboration.

### Strengths and limitations

There are three strengths to this study. Firstly, this study contributes qualitative and descriptive self-reported evidence regarding the perceived impact of simulation-based experiential learning on undergraduates studying global health and other health-based and science-based disciplines preparedness for real-world challenges and leadership roles. While our findings are based solely on post-simulation self-reported measures and do not include objective pre/post testing or longitudinal follow-up, they provide insight into how participants perceive the value of such simulations in their educational and professional development. Second, the study’s use of a WHA simulated environment provided a unique, immersive setting that closely mirrors the complexities of global health diplomacy and decision-making. This realism adds a layer of practical experience to the application of theoretical knowledge, helping to evaluate participants’ problem-solving and leadership skills in a manner that more traditional, classroom-based learning methodologies could not replicate. Thirdly, the sample includes students from several academic programs within one institution, which provided multiple academic perspectives. However, we do not claim that the sample is broadly representative or offers broad cultural diversity. This limitation should be considered when interpreting the findings, as they may not generalize beyond this institutional and disciplinary context.

One limitation is the potential for self-assessment bias in the quantitative data, as participants may have overestimated or underestimated their competencies and experiences, which could affect the reliability of the data. To address this limitation regarding potential self-assessment bias, we designed the survey with a five-point scale for self-evaluation.

Another limitation of our study is its primary reliance on post-simulation self-assessment surveys and qualitative reflections to capture participants’ perceptions following their participation in the WHA SIM. These self-reported measures do not constitute formal summative assessments of performance. To strengthen future evaluations, we recommend incorporating both formative assessment techniques (such as real-time feedback sessions and reflective writing tasks) and validated summative assessments to objectively measure participant learning and skill development. These additional assessment strategies could provide immediate insights to refine and improve the simulation for future cohorts.Additionally, the absence of specific recruitment numbers at each stage is a limitation, as the recruitment messages were sent to a confidential student distribution list managed by university administrators. The simultaneous implementation of recruitment methods also made it difficult to attribute specific numbers to each method. To mitigate this, future studies could consider launching recruitment efforts in stages, if time permits, to gather specific numbers for each method. This approach would provide a clearer picture of participant outreach and ensure a more transparent recruitment process.

### Implications for teaching practice

Our research outlines four key strategies to enhance simulation-based learning in global health: enhancing curriculum design, fostering competence and confidence, emphasizing interdisciplinary collaboration, and encouraging lifelong learning (Table 2). Incorporating simulation-based learning into curricula fosters key competencies – communication, leadership, and problem-solving – essential for global health careers. Fostering confidence is crucial, especially given participants’ reported discomfort with specific professional interactions, such as engaging leadership. Targeted training using role-playing and feedback may boost confidence. Interdisciplinary collaboration enhances students’ ability to engage with diverse professionals, fostering richer knowledge exchange. Finally, encouraging lifelong learning equips students to adapt to evolving global health challenges, technologies, and contexts.

**Table 2:** Strategies for enhancing simulation-based experiential learning.

Strategy	Description
Enhancing curriculum design	This involves incorporating simulation-based experiential learning into student curricula to allow the development of skills through the application of knowledge, fostering the development of skills, and offering valuable opportunities for self-reflection on strengths and areas for continued growth.
Fostering competence and confidence	This involves empowering students to engage more actively. This approach will enhance their ability to make informed decisions, enhance problem-solving skills, and prepare them to handle complex issues by supporting their ability to adapt to the real-world global health context.
Emphasizing interdisciplinary collaboration	This involves incorporating diverse academic perspectives and methodologies, encouraging students to think beyond traditional boundaries and understand the multifaceted nature of global health challenges. Engaging with varied disciplines gives students a holistic view essential for effective problem-solving in a complex, interconnected world.
Encouraging lifelong learning	This involves helping students develop a growth mindset, prompting them to continually seek knowledge and improve their skills. This will allow them to adapt to changing scenarios, apply new insights to future challenges, and enhance their ability to tackle challenges with resilience and innovation.

### Implications for academic institutions

Our findings suggest academic institutions would benefit from expanding simulation-based experiential learning within global health and cognate programs. Simulations, such as the WHA SIM, offer benefits that are difficult to achieve through traditional learning methods or classroom settings, helping students develop soft skills vital for global health leadership. Institutions might invest in comprehensive simulation programs, partnerships with global health organizations, and faculty development in simulation facilitation in order to maximize the benefits of simulation-based experiential learning activities. Longitudinal tracking of graduates could provide data to refine these educational approaches, ensuring graduates are equipped to meet complex global health demands.

### Future research

Future research should examine the long-term influence of simulation-based experiential learning on global health students’ careers and lifelong learning. Longitudinal studies following participants could clarify whether the perceived benefits of simulation-based learning translate into actual career outcomes, professional development, career decisions, leadership roles, and sustained engagement in global health work. Comparative studies evaluating simulation against alternative educational methods would also provide insights into their relative advantages and limitations, informing curricular decisions.

## Conclusions

The WHA SIM emerges as a valuable pedagogical tool for preparing global health students for future leadership and collaborative roles. This study highlighted participants’ self-reported perceptions, offering important insights into their experiences and self-assessed skill development - crucial indicators in educational research. The WHA SIM simulates a realistic environment fostering skills appraisal, enhancing understanding of global health careers, and nurturing leadership development in a real-world context. These findings underscore its potential to support and improve students’ readiness for professional roles in global health.

### Contributions to the literature

This study contributes to the burgeoning literature on experiential learning in health sciences, particularly in the context of global health governance. By employing a descriptive sequential exploratory approach, it provides insights into students’ self-reported confidence and reflections on a simulation-based learning platform. These findings may inform the development of future simulation-based educational initiatives, emphasizing the importance of participant perceptions and experiences rather than measured effectiveness.

## Supporting information

S1 Table Characteristics of research participants. (DOCX)

S2 Table Summary of findings table. (DOCX)
