# Supplementary material for: Using a World Health Assembly simulation to explore undergraduate students’ perceptions and confidence in analyzing complex global health challenges: A mixed-methods evaluation
Source: PLOS Glob Public Health. 2025 Nov 3;5(11):e0002792. doi: 10.1371/journal.pgph.0002792 (PMC12582484; doi:10.1371/journal.pgph.0002792)
Supplement: S1 Table — (DOCX) [file pgph.0002792.s001.docx]

**S1 Table**

**Table 1.** Characteristics of Research Participants

| **Study Design** | **# of Participants (n)** | **Organizational Affiliations** | **Program of Study** | **Academic Year** | **Sex** |
| --- | --- | --- | --- | --- | --- |
| **Survey** | (n=39) | York University  ​​ (n= 39)​  ​​​ | ​​​Global Health ​  ​​​(n=34)​​  ​​​Health Management (n=3)​​  ​​​Biology ​​  ​​​(n=1)​​​​  ​​​Biomedical Science ​​  ​​(n=1)​ | ​​​​Fourth-year ​​  ​​​(n= 21)​​  ​​​Third-year (n= 6)​​  ​​​Second-year ​​  ​​​(n= 3)​​  ​​​First-year​​  ​​​(n= 3)​​  ​​​Other ​​  ​​​(n= 6)​​​​​​​​​ | ​​34​ F  ​​5​ M |
| **Semi-structured interviews** | (n=18) | York University  ​​(n= 18)​  ​​​ | ​​Global Health​​​  (n=17)  ​​Health Management  (n = 1) | Fourth-year (n=1​1​​)  Third-year  (n=3)  Second-year (n= 1)  Other  (n=​3​​) | ​​14 F  4 M |
