# Supplementary material for: Using a World Health Assembly simulation to explore undergraduate students’ perceptions and confidence in analyzing complex global health challenges: A mixed-methods evaluation
Source: PLOS Glob Public Health. 2025 Nov 3;5(11):e0002792. doi: 10.1371/journal.pgph.0002792 (PMC12582484; doi:10.1371/journal.pgph.0002792)
Supplement: S2 Table — (DOCX) [file pgph.0002792.s002.docx]

**S2 Table:**

**Table 1.** Summary of findings table

| ​ **Participants views and experiences in simulation-based experiential learning.** | | **N (%)** |
| --- | --- | --- |
| **Involvement in simulation-based learning** | *Yes* | ​​13 (33.33)​ |
|  | *No* | ​​*20 (51.28)*​ |
|  | *Not sure* | ​​*6 (15.38)*​ |
| **Awareness of what simulation-based experiential learning is** | *Yes* | ​​26 (66.67)​ |
|  | *No* | ​​*7 (17.95)*​ |
|  | *Somewhat* | ​​*6 (15.38)*​ |
| **Awareness of what a World Health Organization World Health Assembly is about** | *Never heard of it before* | ​​2 (5.13)​ |
|  | *Heard of it before* | ​​*30 (76.92)*​ |
|  | *Partially aware of what it is* | ​​*7 (17.95)*​ |
| **Belief that simulation-based learning is a necessary aspect of learning** | *Yes* | ​​23 (58.97)​ |
|  | *No* | ​​*1 (2.56)*​ |
|  | *Not sure* | ​​*15 (38.46)*​ |
| **Incorporation of any form of simulation-based learning during degree completion** | *Yes* | ​​15 (38.46)​ |
|  | *No* | ​​*16 (41.03)*​ |
|  | *Not sure* | ​​*8 (20.51)*​ |
| **Views on how effective simulation-based learning in preparing for real-world experiences** | *Very effectively* | ​​19 (48.72)​ |
|  | *Somewhat effectively* | ​​*20 (51.28)*​ |
|  | *Very ineffectively* | ​​*0*​ |
|  | *Somewhat ineffectively* | ​​*0*​ |
|  | *Neither effectively nor ineffectively* | ​​*0*​ |

| **Participants Levels of Comfort and Confidence in Key Practical Skills.** | | **N (%)** |
| --- | --- | --- |
| **Confidence in communicating with others (e.g., colleagues)** | *Strongly agree* | *16 (41.03)* |
|  | *Agree* | *​​20 (51.28)​* |
|  | *Disagree* | *​​3 (7.69)​* |
|  | *Strongly disagree* | *​​0​* |
| **Comfort in communicating with leadership (e.g., decision-makers, professors, technical experts)** | *Strongly agree* | *​​10 (25.64)​* |
|  | *Agree* | *​​16 (41.03)​* |
|  | *Disagree* | *​​12 (30.77)​* |
|  | *Strongly disagree* | *​​1 (2.56)​* |
| **Comfort in delegating tasks to team members** | *Strongly agree* | *​​11 (28.21)​* |
|  | *Agree* | *​​24 (61.54)​* |
|  | *Disagree* | *​​4 (10.26)​* |
|  | *Strongly disagree* | *​​0​* |
| **Difficulty in prioritizing tasks** | *Strongly agree* | *​​2 (5.13)​* |
|  | *Agree* | *​​13 (33.33)​* |
|  | *Disagree* | *​​19 (48.72)​* |
|  | *Strongly disagree* | *​​5 (12.82)​* |
| **Confidence in problem-solving** | *Strongly agree* | *​​11 (28.95)​* |
|  | *Agree* | *​​22 (57.89)​* |
|  | *Disagree* | *​​5 (13.16)​* |
|  | *Strongly disagree* | *​​0​* |
| **Opportunities to practice time management skills** | *Strongly agree* | *​​16 (41.03)​* |
|  | *Agree* | *​​22 (56.41)​* |
|  | *Disagree* | *​​1 (2.56)​* |
|  | *Strongly disagree* | *​​0​* |
| **Opportunities to critically analyze and problem solve** | *Strongly agree* | *​​13 (33.33)​* |
|  | *Agree* | *​​24 (61.54)​* |
|  | *Disagree* | *​​2 (5.13)​* |
|  | *​​Strongly disagree​* | *​​​0​​* |
| **​​Comfort in asking for help​** | *​​Strongly agree​* | *​​​14 (35.90)​​* |
|  | *​​Agree​* | *​​​18 (46.15)​​* |
|  | *​​Disagree​* | *​​​7 (17.95)​​* |
|  | *​​Strongly disagree​* | *​​​0​​* |
| **​​Confidence in using current evidence to solve global health problems ​** | *​​Strongly agree​* | *​​​17 (43.59)​​* |
|  | *​​Agree​* | *​​​21 (53.85)​​* |
|  | *​​Disagree​* | *​​​1 (2.56)​​* |
|  | *​​Strongly disagree​* | *​​​0​​* |
| **​​Readiness for professional practice after graduation ​** | *​​Strongly agree​* | *​​​8 (20.51)​​* |
|  | *​​Agree​* | *​​​18 (46.15)​​* |
|  | *​​Disagree​* | *​​​10 (25.64)​​* |
|  | *​​Strongly disagree​* | *​​​3 (7.69)​​* |

*Confidence in problem-solving: A participant did not answer this question, resulting in n=38.*

| ​​**Participants perception of the role of simulation-based learning in preparing for real-world career skills** | | **N (%)** |
| --- | --- | --- |
| **Orientation to work environment** | *Very helpful* | ​​27 (69.23)​ |
|  | *A little helpful* | ​​*4 (10.26)*​ |
|  | *Somewhat helpful* | ​​*8 (20.51)*​ |
|  | *Not helpful* | ​​*0*​ |
|  | *Not applicable* | ​​*0*​ |
| **Ability to tackle complex global health challenges** | *Very helpful* | ​​29 (74.36)​ |
|  | *A little helpful* | ​​*2 (5.13)*​ |
|  | *Somewhat helpful* | ​​*7 (17.95)*​ |
|  | *Not helpful* | ​​*0*​ |
|  | *Not applicable* | ​​*1 (2.56)*​ |
| **Managing workloads (e.g., organizing, prioritizing)** | *Very helpful* | ​​21 (53.85)​ |
|  | *A little helpful* | ​​*3 (7.69)*​ |
|  | *Somewhat helpful* | ​​*15 (38.46)*​ |
|  | *Not helpful* | ​​*0*​ |
|  | *Not applicable* | ​​*0*​ |
| **Confidence in delegation, knowledge and critical thinking** | *Very helpful* | ​​29 (74.36)​ |
|  | *A little helpful* | ​​*1 (2.56)*​ |
|  | *Somewhat helpful* | ​​*8 (20.51)*​ |
|  | *Not helpful* | ​​*0*​ |
|  | *Not applicable* | ​​*1 (2.56)*​ |
| **​​Understanding role expectations and legal/regulatory issues (e.g., autonomy, more responsibility)​** | *Very helpful* | ​​27 (69.23)​ |
|  | *A little helpful* | ​​*1 (2.56)*​ |
|  | *Somewhat helpful* | ​​*9 (23.08)*​ |
|  | *Not helpful* | ​​*0*​ |
|  | *Not applicable* | ​​*2 (5.13)*​ |
| **​​Confidence in communication with others including decision-makers** | *Very helpful* | ​​25 (64.10)​ |
|  | *A little helpful* | ​​*2 (5.13)*​ |
|  | *Somewhat helpful* | ​​*11 (28.21)*​ |
|  | *Not helpful* | ​​​*0*​​ |
|  | ​​*Not applicable* ​ | ​​​*1 (2.56)*​​ |
| **​​​Knowing when to ask for assistance ​​** | ​​*Very helpful*​ | ​​​17 (43.59)​​ |
|  | ​​*A little helpful*​ | ​​​*6 (15.38)*​​ |
|  | ​​*Somewhat helpful*​ | ​​​*16 (41.03)*​​ |
|  | ​​*Not helpful*​ | ​​​*0*​​ |
|  | ​​*Not applicable* ​ | ​​​*0*​​ |
| **​​​Respecting diverse cultural perspectives and opinions ​​** | ​​*Very helpful*​ | ​​​22 (56.41)​​ |
|  | ​​*A little helpful*​ | ​​​*4 (10.26)*​​ |
|  | ​​*Somewhat helpful*​ | ​​​*12 (30.77)*​​ |
|  | ​​*Not helpful*​ | ​​​*1 (2.56)*​​ |
|  | ​​*Not applicable* ​ | ​​​*0*​​ |
| **​​​Documenting and using technology proficiently​​** | ​​*Very helpful*​ | ​​​21 (53.85)​​ |
|  | ​​*A little helpful*​ | ​​​*4 (10.26)*​​ |
|  | ​​*Somewhat helpful*​ | ​​​*11 (28.21)*​​ |
|  | ​​*Not helpful*​ | ​​​*0*​​ |
|  | ​​*Not applicable* ​ | ​​​*3 (7.69)*​​ |
| **​​Working in collective decision-making settings** | ​​*Very helpful*​ | ​​​29 (76.32)​​ |
|  | ​​*A little helpful*​ | ​​​*2 (5.26)*​​ |
|  | ​​*Somewhat helpful*​ | ​​​*6 (15.79)*​​ |
|  | ​​*Not helpful*​ | ​​​*0*​​ |
|  | ​​*Not applicable* ​ | ​​​*1 (2.63)*​​ |
| **Enhancing your leadership skills** | ​​*Very helpful*​ | ​​​30 (78.95)​​ |
|  | ​​*A little helpful*​ | ​​​*0*​​ |
|  | ​​*Somewhat helpful*​ | ​​​*5 (13.16)*​​ |
|  | ​​*Not helpful*​ | ​​​*1 (2.63)*​​ |
|  | ​​*Not applicable* ​ | ​​​*2 (5.26)*​​ |

*Working in collective decision-making settings: A participant did not answer this question, resulting in n=38.*

*Enhancing your leadership skills: A participant did not answer this question, resulting in n=38.*
